# Cumulative Protective Effect of Melatonin and Indole-3-Propionic Acid against KIO_3_—Induced Lipid Peroxidation in Porcine Thyroid

**DOI:** 10.3390/toxics9050089

**Published:** 2021-04-21

**Authors:** Paulina Iwan, Jan Stepniak, Malgorzata Karbownik-Lewinska

**Affiliations:** 1 Department of Oncological Endocrinology, Medical University of Lodz, 7/9 Zeligowski St., 90-752 Lodz, Poland; paulina.iwan@op.pl (P.I.); jan.stepniak@umed.lodz.pl (J.S.); 2 Polish Mother’s Memorial Hospital—Research Institute, 281/289 Rzgowska St., 93-338 Lodz, Poland

**Keywords:** melatonin, indole-3-propionic acid, potassium iodate, KIO_3_, lipid peroxidation, thyroid cancer, antioxidant, salt iodization

## Abstract

Iodine deficiency is the main environmental factor leading to thyroid cancer. At the same time iodine excess may also contribute to thyroid cancer. Potassium iodate (KIO_3_), which is broadly used in salt iodization program, may increase oxidative damage to membrane lipids (lipid peroxidation, LPO) under experimental conditions, with the strongest damaging effect at KIO_3_ concentration of ~10 mM (corresponding to physiological iodine concentration in the thyroid). Melatonin and indole-3-propionic acid (IPA) are effective antioxidative indoles, each of which protects against KIO_3_-induced LPO in the thyroid. The study aims to check if melatonin used together with IPA (in their highest achievable in vitro concentrations) reveals stronger protective effects against KIO_3_-induced LPO in porcine thyroid homogenates than each of these antioxidants used separately. Homogenates were incubated in the presence of KIO_3_ (200; 100; 50; 25; 20; 15; 10; 7.5; 5.0; 2.5; 1.25; 0.0 mM) without/with melatonin (5 mM) or without/with IPA (10 mM) or without/with melatonin + IPA, and then, to further clarify the narrow range of KIO_3_ concentrations, against which melatonin + IPA reveal cumulative protective effects, the following KIO_3_ concentrations were used: 20; 18.75; 17.5; 16.25; 15; 13.75; 12.5; 11.25; 10; 8.75; 7.5; 0.0 mM. Malondialdehyde + 4-hydroxyalkenals (MDA + 4-HDA) concentration (LPO index) was measured spectrophotometrically. Protective effects of melatonin + IPA were stronger than those revealed by each antioxidant used separately, but only when LPO was induced by KIO_3_ in concentrations from 18.75 mM to 8.75 mM, corresponding to physiological iodine concentration in the thyroid. In conclusion, melatonin and indole-3-propionic acid exert cumulative protective effects against oxidative damage caused by KIO_3_, when this prooxidant is used in concentrations close to physiological iodine concentrations in the thyroid. Therefore, the simultaneous administration of these two indoles should be considered to prevent more effectively oxidative damage (and thereby thyroid cancer formation) caused by iodine compounds applied in iodine prophylaxis.

## 1. Introduction

Free radicals are highly reactive transient molecules, which have an odd number of electrons and are generated in vivo as byproducts of normal metabolism [1,2]. Reactive oxygen species (ROS) include both oxygen radicals (e.g., superoxide anion radical (O_2_^•−^), hydroxyl radical (•OH), and hydroperoxyl radical (•OOH)) and certain nonradical oxidizing agents (i.e., hydrogen peroxide (H_2_O_2_), peroxynitrite anion (ONOO^−^), hypochlorous acid (HOCl) and ozone (O_3_)) easily converted into radicals [1,2]. Under physiological conditions, there is a balance between beneficial and harmful effects of free radicals, which is essential for the survival of organisms and their health [1,2,3]. Any imbalance between these processes may result in different pathological conditions. However, modulation of oxidative stress can serve as a strategy against diseases, cancer included [3].

Oxidative reactions occur practically in all tissues and organs, including thyroid gland, in which ROS play a particular role. This is due to the fact that different factors, such as H_2_O_2_, iron or iodine, are indispensable for thyroid hormone synthesis [4]. For this reason, thyroid gland is characterized by high level of oxidative stress, which—in response to additional oxidative abuse caused by exogenous or endogenous prooxidants—may lead to different thyroid diseases, including cancer [5].

Numerous evidence suggest that environmental factors, including endocrine disruptors, can contribute to thyroid cancer [6]. One of the major risk factors for goiter and, consequently, for thyroid cancer, is iodine deficiency [7]. Moreover, correction of iodine deficiency decreases the prevalence of goiter [8] and might shift thyroid cancer subtypes toward less malignant forms [7]. On the other hand, iodine excess may lead to thyroiditis, thyroid dysfunction, and also to papillary thyroid cancer [9].

To eliminate iodine deficiency, iodized salt is used in most countries in iodine prophylaxis. Programs of salt iodization are based on the use of either potassium iodide (KI) or potassium iodate (KIO_3_) [10]. It is known that these two main iodine compounds have different pro- and antioxidative properties. KIO_3_, in contrast to KI, is the oxidant and thereby may react easily with oxidizable substances [11]. It has been documented recently that KIO_3_ and KI reveal different in vitro effects on oxidative damage to macromolecules in the thyroid [12,13,14,15]. In these studies, KIO_3_ did not reveal any protective effects; instead, it damaged by itself membrane lipids with the strongest damaging effect observed at concentrations of 10 mM [12] or of 15 mM [14,15], which both correspond to physiological iodine concentration in the thyroid [16,17,18]. However, KIO_3_ has still GRAS (“generally recognized as safe”) status given by FDA [19].

The increased oxidative stress can be diminished by antioxidants. Indole substances belong to very effective antioxidants. The most important representative of indole substances is melatonin (5-methoxy-*N*-acetyltryptamine). Melatonin is mainly produced by the pineal gland; it is a tryptophan metabolite which is repeatedly documented to reduce oxidative stress [20]. Melatonin effectively scavenges different free radicals and ROS; it is one of the strongest scavengers of •OH [21]. Additionally, its metabolites (*N*1-acetyl-*N*2-formyl-5-methoxykynuramine (AFMK), *N*-acetyl-5-methoxyknuramine (AMK), and cyclic-3-hydroxymelatonin (c3OHM)) have been also found to protect cells from ROS [22,23,24]. It has been demonstrated that one melatonin molecule has the capacity to scavenge up to 10 molecules of ROS [22]. Numerous studies revealed protective effects of melatonin against oxidative damage to macromolecules caused by potential carcinogens, as it has been summarized by us previously [25,26].

The main physiological function of melatonin is to regulate circadian rhythm [27]. Melatonin also causes positive effects on other physiological processes, such as for example bone formation, body mass regulation, reproduction, regulation of immune system and cardiovascular system, as well as it serves as a pharmacological agent [28]. Doses between 1 mg and 6 mg appear to be effective for improving sleep in older adults [29]. In clinical trials investigating anxiety, melatonin in doses varied from 3 to 10 mg probably reduced preoperative anxiety in adults, which is potentially clinically relevant [30]. Available studies show, that short-time use of melatonin, even in very high doses, is safe. Some randomized clinical studies revealed only mild side effects during long-time administration of this drug, comparable to placebo treatment, i.e., sleepiness, dizziness, headache or nausea [31].

Indole-3-propionic acid (IPA), an indole substance possessing a chemical structure similar to that of melatonin, is another effective antioxidant. Similar to melatonin, it scavenges effectively •OH [32]. Indole-3-propionic acid has been documented to protect against oxidative damage to membranes caused by such potential carcinogens as potassium bromate, iron or chromium [33,34,35,36]. Its potential favorable properties in humans include, among others, therapeutic strategy for Alzheimer disease [37].

Melatonin has been shown to prevent experimentally-induced oxidative damage to macromolecules in the thyroid gland [33,38,39]. This indole substance also inhibits thyroid growth and thyroid function [40]. As melatonin is confirmed to prevent the increased oxidative damage in the thyroid and to inhibit growth processes in this gland, it should be considered as a potential protective agent against thyroid cancer.

In our previous studies we have observed that not only melatonin [14], but also IPA [15] are able—in concentration-dependent manner—to reduce oxidative damage to membrane lipids caused by KIO_3_, when this compound was used in doses close to physiological iodine concentrations in the thyroid. In the present study we decided to check if melatonin used together with IPA (in their highest achievable in vitro concentrations resulting from their limited solubility) reveals stronger protective effects against KIO_3_-induced oxidative damage to membrane lipids in porcine thyroid homogenates comparing to protective effects of each antioxidant used separately.

## 2. Materials and Methods

### 2.1. Chemicals

Potassium iodate (KIO_3_), melatonin and indole-3-propionic acid (IPA) were purchased from Sigma (St. Louis, MO, USA). The ALDetect Lipid Peroxidation Assay Kit was obtained from Enzo Life Sciences, Inc. (Zandhoven, Belgium). All the used chemicals were of analytical grade and came from commercial sources.

### 2.2. Animals

Porcine thyroids were collected from eighteen (18) animals at a slaughter-house, frozen on solid CO_2_ and stored at −80° until assay. Each experiment was repeated three times. Therefore, three tissue pools were prepared, with six (6) thyroid glands used for each homogenate pool.

### 2.3. Assay of Lipid Peroxidation

Thyroid tissue was homogenized in ice cold 20 mM Tris-HCl buffer (pH 7.4) (10%, *w*/*v*) and then incubated for 30 min at 37° in the presence of examined substances.

In Experiment I thyroid homogenates were incubated in the presence of KIO_3_ (200; 100; 50; 25; 20; 15; 10; 7.5; 5.0; 2.5; 1.25; 0.0 mM) without any antioxidant or with addition of either melatonin (5 mM) or IPA (10 mM) or both (melatonin 5 mM + IPA 10 mM).

In Experiment II, to further clarify the range of KIO_3_ concentrations, against which melatonin + IPA reveal cumulative effects, the following KIO_3_ concentrations were used: 20; 18.75; 17.5; 16.25; 15; 13.75; 12.5; 11.25; 10; 8.75; 7.5; 0.0 mM. Therefore, thyroid homogenates were incubated in the presence of KIO_3_ (in above concentrations) without any antioxidant or with addition of either melatonin (5 mM) or IPA (10 mM) or both (melatonin 5 mM + IPA 10 mM).

The concentrations of KIO_3_ [12,14,15], of melatonin and of IPA [14,15,33,38] were chosen on the basis of the results of our previous studies; the highest achievable concentrations of melatonin and IPA resulting from their limited solubility were used.

The reactions were stopped by cooling the samples on ice.

### 2.4. Measurement of Lipid Peroxidation Products

The concentrations of malondialdehyde + 4-hydroxyalkenals (MDA + 4-HDA), as an index of lipid peroxidation, were measured in thyroid homogenates, with the ALDetect Lipid Peroxidation Assay Kit. The homogenates were centrifuged at 5000× *g* for 10 min at 4°. After obtaining supernatant, each experiment was carried out in duplicate. The supernatant (200 μL) was mixed with 650 μL of a methanol: acetonitrile (1:3, *v*/*v*) solution, containing a chromogenic reagent, N-methyl-2-phenylindole, and vortexed. Following the addition of 150 μL of methanesulfonic acid (15.4 M), the incubation was carried out at 45° for 40 min. The reaction between MDA + 4-HDA and N-methyl-2-phenylindole yields a chromophore, which is spectrophotometrically measured at the absorbance of 586 nm, using a solution of 10 mM 4-hydroxynonenal as the standard. The level of lipid peroxidation is expressed as the amount of MDA + 4-HDA (nmol) per mg protein. Protein was measured using Bradford’s method, with bovine albumin as the standard [41].

### 2.5. Statistical Analyses

The data were statistically analyzed, using a one-way analysis of variance (ANOVA), followed by the Student–Neuman–Keuls’ test, or using an unpaired t-test. Statistical significance was determined at the level of *p* < 0.05. Results are presented as means ± SE.

## 3. Results

**Figure 1:**
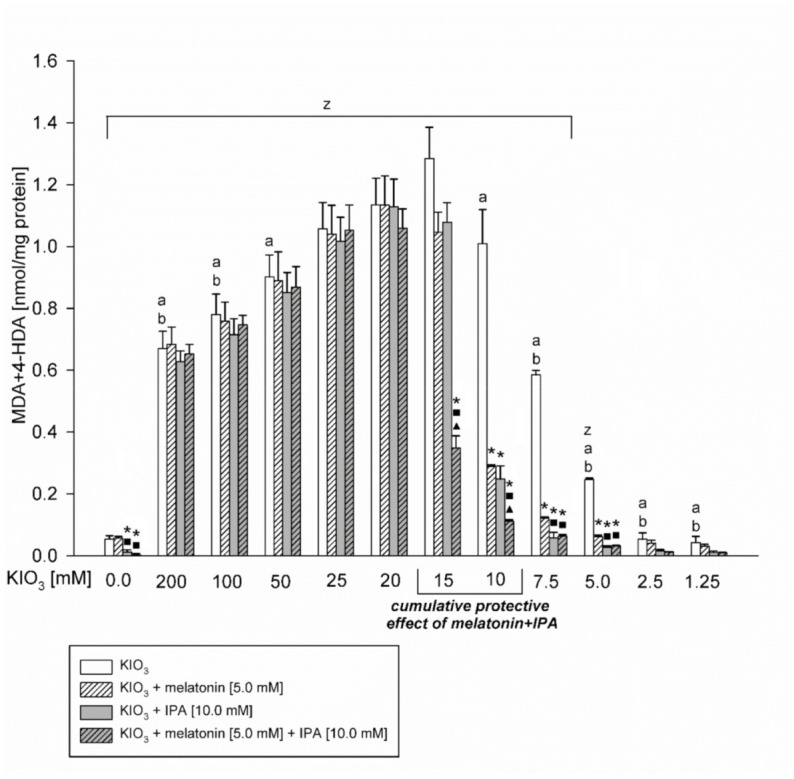
Lipid peroxidation, measured as MDA + 4-HDA level, in porcine thyroid homogenates, incubated in the presence of KIO_3_ (200; 100; 50; 25; 20; 15; 10; 7.5; 5.0; 2.5; 1.25; 0.0 mM) (white bars), or KIO_3_ + melatonin [5 mM] (striped bars), or KIO_3_ + IPA [10 mM] (grey bars), or KIO_3_ + melatonin [5 mM] + IPA [10 mM] (striped grey bars). *—*p* < 0.05 vs. KIO_3_. a—*p* < 0.05 vs. KIO_3_ [15 mM]. b—*p* < 0.05 vs. KIO_3_ [10 mM, 20 mM, and 25 mM]. z—*p* < 0.05 vs. respective control. **■**—***p*** **< 0.05 vs. KIO_3_ in the same concentration + melatonin.** **▲—*p*** **< 0.05 vs. KIO_3_ in the same concentration + IPA.**

In the Experiment I, IPA (10 mM) and melatonin (5 mM), applied separately, reduced KIO_3_-induced lipid peroxidation when this prooxidant was used at concentrations of 10 mM, 7.5 mM or 5.0 mM (Figure 1), which is in line with the results of our previous studies [14,15].

**Figure 2:**
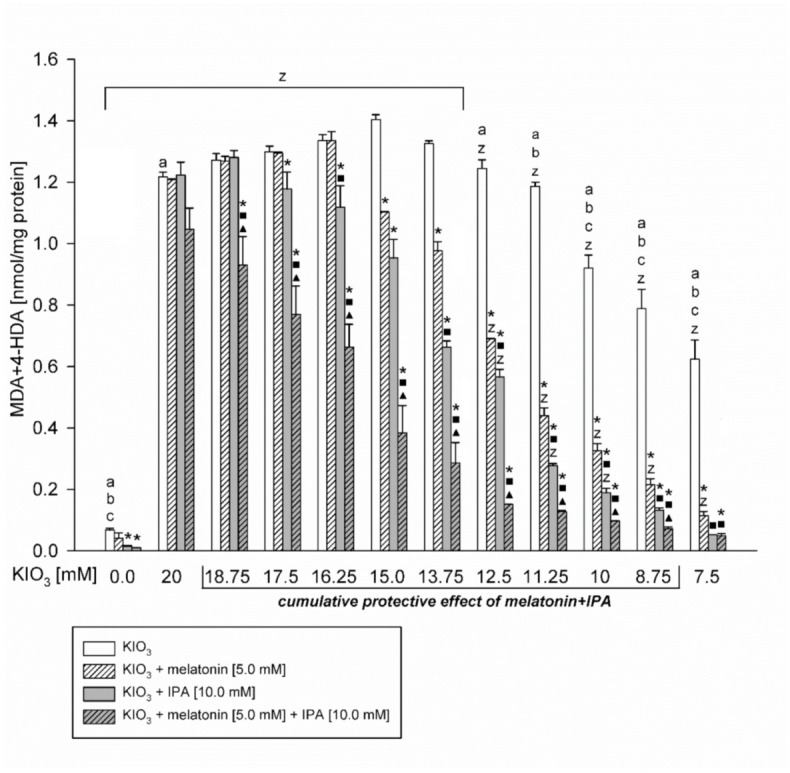
Lipid peroxidation, measured as MDA + 4-HDA level, in porcine thyroid homogenates, incubated in the presence of KIO_3_ (20; 18.75; 17.5; 16.25; 15; 13.75; 12.5; 11.25; 10; 8.75; 7.5; 0.0 mM) (white bars), or KIO_3_ + melatonin [5 mM] (striped bars), or KIO_3_ + IPA [10 mM] (grey bars), or KIO_3_ + melatonin [5 mM] + IPA [10 mM] (striped grey bars). *—*p* < 0.05 vs. KIO_3_. a—*p* < 0.05 vs. KIO_3_ [15 mM]. b—*p* < 0.05 vs. KIO_3_ [16.25 mM]. c—*p* < 0.05 vs. KIO_3_ [13.75 mM]. z—*p* < 0.05 vs. respective control. **■—*p* < 0.05 vs. KIO_3_ in the same concentration + melatonin [5 mM]. ▲—*p* < 0.05 vs. KIO_3_ in the same concentration + IPA [10 mM]**.

However, in Experiment II with the use of additional concentrations of KIO_3_, IPA revealed protective effects against higher concentration of KIO_3_ (16.25 mM) than melatonin did (KIO_3_ in the concentration of 15 mM) (Figure 2). Additionally, protective effects of IPA were stronger than those of melatonin against oxidative damage caused by KIO_3_ at concentrations of 13.75 mM or lower (Figure 2).

The most important observation is that melatonin used together with IPA revealed stronger protective effects than each of these antioxidants used separately, but only when LPO was induced by KIO_3_ in concentrations of 15 mM and 10 mM (Experiment I, Figure 1) or in the range of concentrations from 18.75 mM to 8.75 mM (Experiment II, Figure 2). These cumulative protective effects of melatonin + IPA are especially evident at higher KIO_3_ concentrations, i.e., 18.75 mM and 17.5 mM, against which no protection was seen when either melatonin or IPA were used separately.

It has been also observed that melatonin did not change the basal LPO level, whereas IPA or IPA + melatonin decreased the basal LPO level (Figure 1 and Figure 2).

## 4. Discussion

This study is a continuation of our research on the antioxidative properties of melatonin and other indole substances. Taking into account properties of these substances we decided to use concomitantly two effective antioxidants—melatonin and IPA—in their highest achievable in vitro concentrations, i.e., 5 mM for melatonin and 10 mM for IPA, to evaluate their cumulative effect against oxidative damage caused by KIO_3_.

Because iodate has been conferred GRAS status by FDA [19,42] and due to its greater chemical stability comparing to iodide, most health authorities recommend using preferentially the former iodine compound as an additive to salt for correcting iodine deficiency [43]. Iodate was tested for its potential toxicity, but it has not been confirmed till now in humans. However, taking into account that iodic acid (HIO_3_) belongs to the class of oxohalogen acids, has similar chemical structure to that one of KBrO_3_ (known potential carcinogen belonging to the group 2B according to IARC [44]) and reveals prooxidative effects documented in our previous studies [12,14,15], it cannot be excluded that this compound may be potentially dangerous.

Currently, despite the worldwide strategies for the prevention and control of iodine deficiency, it is still a widespread public health issue, especially in pregnant women. Severe iodine deficiency may be associated with many adverse effects, such as the increased risk of pregnancy loss and infant mortality, neonatal hypothyroidism, cretinism and neuropsychomotor retardation [45,46]. Moreover, as it was mentioned above, iodine deficiency may lead to goiter—a risk factor for thyroid cancer [7]. As KIO_3_ is broadly used for salt iodization and as potential toxicity of KIO_3_ has been observed in experimental studies, it is justified to look for safe factors, which can prevent any damage potentially caused by KIO_3_. For this reason, we continue our research on the antioxidative properties of melatonin and other indole substances with relation to protection against oxidative damage to membrane lipids caused by KIO_3_.

In the present study either melatonin or IPA decreased lipid peroxidation induced by KIO_3_, what is in agreement with our previous observations [14,15]. The most important observation is, however, that melatonin used together with IPA revealed even stronger protective effects than each of these antioxidants used separately. It should be stressed that the protective effects of either melatonin or IPA [14,15] as well as of both indole substances used simultaneously (the present study) were observed only when KIO_3_ was applied in concentrations (from 10 mM to 7.5 mM in [14,15]; from 18.75 mM to 8.75 mM in the present study) corresponding to physiological iodine concentration in the thyroid, which obviously result from recommended iodine supply. The physiological iodine concentration in rat and human thyroid was calculated to be approx. 9.0 mM [16,17,18] (being in the range of KIO_3_ concentrations from 18.75 mM to 8.75 mM). Taking into account similarity between porcine and human thyroid, it can be assumed that concentration of iodine in porcine thyroid is similar.

Antioxidative effects of melatonin have been known for a long time [21,24,28]. These effects were observed not only in the thyroid gland [38], but also in other tissues, both in vivo and in vitro experiments [47,48]. Mechanisms by which melatonin protects against LPO are as follows: melatonin stimulates antioxidative enzymes, i.e., glutathione peroxidase, glutathione reductase, superoxide dismutase and catalase, upregulates synthesis of glutathione (another intracellular antioxidant) and cooperates with free radical scavengers [20,24]. Moreover, melatonin is able to detoxify practically all free radicals and reactive species, such as •OH [21], nitric oxide and ONOO^−^, and to suppress nitric oxide synthase [20,24]. Furthermore, metabolites of melatonin (AMK, AFMK and c3OHM) can protect against oxidative damage, as all three are highly effective scavengers of the devastatingly reactive •OH, and c3OHM is highly effective in scavenging the •OOH [22,23,24].

Melatonin is regarded as the strongest known antioxidant, but there are available studies, which showed superiority of IPA over melatonin [49]. IPA, similar to melatonin, is an endogenous electron donor that detoxifies the •OH, quenches the O_2_^•-^ and acts synergistically with glutathione [32]. Its side chain cannot be decarboxylated, and thus, unlike other indoles, it cannot be converted to a reactive prooxidant intermediate [50].

Both substances, melatonin and IPA, are recognized as safe and do not reveal any adverse effects [31,37].

We proved, that IPA and melatonin, used together in very high doses, intensified antioxidative effect, at least under in vitro conditions. Therefore, they can be used together, when stronger protective action is expected but none of them can be used separately in higher dose due to their limited solubility.

As it was mentioned in the Introduction, exogenous melatonin is applied therapeutically in doses between 2 and 10 mg. In available studies the highest dose of melatonin used in clinical trials was 25 mg [51]. The intravenous administration of melatonin in a dose of 25 mg resulted in blood concentration of ~7.52 × 10^5^ pg/mL [51]. In another study melatonin used in a dose of 10 mg intravenously resulted in blood concentration of ~3.9 × 10^5^ pg/mL and when used orally, in concentration of ~3.5 × 10^3^ pg/mL [52]. Relating these concentrations to those used by us (5 mM of melatonin is equivalent to ~1.16 × 10^9^ pg/mL) it can be concluded that the concentrations used in the present experiment exceed the standard doses by several orders of magnitude. Unfortunately, similar studies with IPA have not been performed. It should be stressed, that our results concerning protective in vitro effects of melatonin used together with IPA cannot be directly extrapolated into in vivo conditions.

In the context of our results, it is worth recalling that both melatonin and IPA are regarded as interesting chemical compounds with potential properties for use in many fields of medicine. Oncostatic effects of melatonin have been reported in breast cancer, ovarian and endometrial carcinoma, prostate cancer, intestinal tumors or melanoma; melatonin may be used in psychiatric and neurodegenerative disorders (i.e., Alzheimer disease, Parkinson disease, amyotrophic lateral sclerosis), diabetes and metabolic syndrome or sepsis [20,24,28,53,54]. The research currently under way evaluates potential protective effects of melatonin against COVID-19 [55,56,57,58]. Concerning IPA, this indole substance—as it was mentioned above—may be regarded as a potential treatment option for Alzheimer’s disease [37].

The current study is the next one in which we observed antioxidative effects of melatonin [14] and IPA [15] against oxidative damage caused by KIO_3_ used at concentrations close to physiological iodine concentration in the thyroid. However, the current study is the first to document that protective effect of one indole substance can be enhanced by the simultaneous use of another indole substance. The importance of the present finding relies on the fact that due to limited solubility of indole substances it is possible to increase the effectiveness of a chosen substance only by the use of another indole substance. The future studies should focus on to check if the simultaneous use of more than two indole substances can still increase protective effects against experimentally-induced oxidative damage caused by different prooxidants.

The important limitation of our study is that the obtained results cannot be directly extrapolated into in vivo conditions. It is worth mentioning, that in in vivo conditions IO_3_^−^ should be reduced to I^−^ by nonenzymatic reactions before it can become available to the body as iodide [43]. Recent studies showed that in rats even high doses of IO_3_^−^ were completely reduced to I^−^ in vivo within 30 min [59]. The results suggest that IO_3_^−^ may be reduced in the digestive tract before I^−^ enters the blood, but this mechanism is still unexplained [59]. Similar effects were observed in rat homogenates—IO_3_^−^ was reduced to I^−^— in vitro [11]. However, whereas KIO_3_ decreased total antioxidative activity and NADPH concentration in tissues in vitro [11], this effect of KIO_3_ has not been confirmed in vivo, i.e., KIO_3_ did not affect the total antioxidative activity in blood serum and in other tissues [59]. These differences between results obtained in vivo and in vitro require further research to better understand KIO_3_ effects in various conditions. At this moment we can state that presumably, except for the gastrointestinal mucosa, exposure of other tissues (including the thyroid gland) to iodate (after its systemic administration) might be minimal. At the same time, however, it is not excluded that even minimal exposure of prooxidative agent can produce toxic effects.

In our previous studies [14,15] we tried to answer the question, why melatonin and IPA were effective against these concentrations of KIO_3_ which correspond to physiological iodine concentration in the thyroid. We proposed a hypothesis, that during phylogenetical development in mammals, some protective mechanisms have been developed to protect against well recognized toxic agents, to which organisms are potentially endangered for years. However, much higher concentrations of iodine, resulting e.g., from pharmacological treatment, are not a common and physiological state; that is why protective mechanisms have not been developed against these rare conditions. The results of our current study also seem to confirm this hypothesis.

## 5. Conclusions

Melatonin and indole-3-propionic acid exert cumulative protective effect against oxidative damage caused by KIO_3_, when this prooxidant is used in concentrations close to physiological iodine concentrations in the thyroid. Therefore, the simultaneous administration of these two indoles should be considered to prevent more effectively oxidative damage (and thereby thyroid cancer formation) caused by iodine compounds applied in iodine prophylaxis.

## Data Availability

The datasets used and/or analyzed during the current study are available from the corresponding author on reasonable request.

## References

[1] Hunyadi A. (2019). The mechanism(s) of action of antioxidants: From scavenging reactive oxygen/nitrogen species to redox signaling and the generation of bioactive secondary metabolites. Med. Res. Rev..

[2] Schieber M., Chandel N.S. (2014). ROS function in redox signaling and oxidative stress. Curr. Biol..

[3] Gorrini C., Harris I.S., Mak T.W. (2013). Modulation of oxidative stress as an anticancer strategy. Nat. Rev. Drug Discov..

[4] Carvalho D.P., Dupuy C. (2017). Thyroid hormone biosynthesis and release. Mol. Cell Endocrinol..

[5] Karbownik-Lewinska M., Kokoszko-Bilska A. (2012). Oxidative damage to macromolecules in the thyroid—Experimental evidence. Thyroid Res..

[6] Alsen M., Sinclair C., Cooke P., Ziadkhanpour K., Genden E., van Gerwen M. (2021). Endocrine Disrupting Chemicals and Thyroid Cancer: An Overview. Toxics.

[7] Zimmermann M.B., Galetti V. (2015). Iodine intake as a risk factor for thyroid cancer: A comprehensive review of animal and human studies. Thyroid Res..

[8] Szybinski Z., Delange F., Lewinski A., Podoba J., Rybakowa M., Wasik R., Szewczyk L., Huszno B., Gołkowski F., Przybylik-Mazurek E. (2001). A programme of iodine supplementation using only iodised household salt is efficient—The case of Poland. Eur. J. Endocrinol..

[9] Southern A.P., Jwayyed S. Iodine Toxicity, StatPearls. https://www.statpearls.com/ArticleLibrary/viewarticle/40905.

[10] Wu T., Liu G.J., Li P., Clar C. (2002). Iodised salt for preventing iodine deficiency disorders. Cochrane Database Syst. Rev..

[11] Cao X., Ma W., Liu L., Xu J., Wang H., Li X., Wang J., Hang J., Wang Z., Gu Y. (2015). Analysis of potassium iodate reduction in tissue homogenates using high performance liquid chromatography-inductively coupled plasma-mass spectrometry. J. Trace Elem. Med. Biol..

[12] Milczarek M., Stepniak J., Lewinski A., Karbownik-Lewinska M. (2013). Potassium iodide, but not potassium iodate, as a potential protective agent against oxidative damage to membrane lipids in porcine thyroid. Thyroid Res..

[13] Karbownik-Lewinska M., Stepniak J., Milczarek M., Lewinski A. (2015). Protective effect of KI in mtDNA in porcine thyroid: Comparison with KIO3 and nDNA. Eur. J. Nutr..

[14] Iwan P., Stepniak J., Karbownik-Lewinska M. (2019). Melatonin reduces high levels of lipid peroxidation induced by potassium iodate in porcine thyroid. Int. J. Vitam. Nutr. Res..

[15] Iwan P., Karbownik-Lewinska M. (2020). Indole-3-propionic acid reduces lipid peroxidation induced by potassium iodate in porcine thyroid. Interdiscip. Toxicol..

[16] Taurog A., Chaikoff I.L., Feller D.D. (1947). The mechanism of iodine concentration by the thyroid gland: Its non-organic iodine-binding capacity in the normal and propylthiouracil-treated rat. J. Biol. Chem..

[17] Taurog A., Tong W., Chaikoff I.L. (1951). Non-thyroglobulin iodine of the thyroid gland II. Inorganic iodide. J. Biol. Chem..

[18] Tiran B., Karpf E., Tiran A., Lax S., Langsteger W., Eber O., Lorenz O. (1993). Iodine content of thyroid tissue in the Styrian population. Acta Med. Austriaca.

[19] Trumbo P.R. (2016). FDA regulations regarding iodine addition to foods and labeling of foods containing added iodine. Am. J. Clin. Nutr..

[20] Reiter R.J., Mayo J.C., Tan D.X., Sainz R.M., Alatorre-Jimenez M., Qin L. (2016). Melatonin as an antioxidant: Under promises but over delivers. J. Pineal Res..

[21] Tan D.X., Manchester L.C., Esteban-Zubero E., Zhou Z., Reiter R.J. (2015). Melatonin as a Potent and Inducible Endogenous Antioxidant: Synthesis and Metabolism. Molecules.

[22] Tan D.X., Manchester L.C., Terron M.P., Flores L.J., Reiter R.J. (2007). One molecule, many derivatives: A never-ending interaction of melatonin with reactive oxygen and nitrogen species?. J. Pineal Res..

[23] Galano A., Tan D.X., Reiter R.J. (2013). On the free radical scavenging activities of melatonin’s metabolites, AFMK and AMK. J. Pineal Res..

[24] Reiter R.J., Tan D.X., Galano A. (2014). Melatonin: Exceeding expectations. Physiology.

[25] Karbownik M., Reiter R.J. (2002). Melatonin protects against oxidative stress caused by delta-aminolevulinic acid: Implications for cancer reduction. Cancer Invest..

[26] Karbownik M., Lewinski A., Reiter R.J. (2001). Anticarcinogenic actions of melatonin which involve antioxidative processes: Comparison with other antioxidants. Int. J. Biochem. Cell Biol..

[27] Bonmati-Carrion M.A., Arguelles-Prieto R., Martinez-Madrid M.J., Reiter R.J., Hardeland R., Rol M.A., Madrid J.A. (2014). Protecting the melatonin rhythm through circadian healthy light exposure. Int. J. Mol. Sci..

[28] Tordjman S., Chokron S., Delorme R., Charrier A., Bellissant E., Jaafari N., Fougerou C. (2017). Melatonin: Pharmacology, Functions and Therapeutic Benefits. Curr. Neuropharmacol..

[29] Pierce M., Linnebur S.A., Pearson S.M., Fixen D.R. (2019). Optimal Melatonin Dose in Older Adults: A Clinical Review of the Literature. Sr. Care Pharm..

[30] Madsen B.K., Zetner D., Møller A.M., Rosenberg J. (2020). Melatonin for preoperative and postoperative anxiety in adults. Cochrane Database Syst. Rev..

[31] Andersen L.P.H., Gögenur I., Rosenberg J., Reiter R.J. (2015). The Safety of Melatonin in Humans. Clin. Drug Investig..

[32] Poeggeler B., Pappolla M.A., Hardeland R., Rassoulpour A., Hodgkins P.S., Guidetti P., Schwarcz R. (1999). Indole-3-propionate: A potent hydroxyl radical scavenger in rat brain. Brain Res..

[33] Karbownik M., Stasiak M., Zasada K., Zygmunt A., Lewinski A. (2005). Comparison of potential protective effects of melatonin, indole-3-propionic acid, and propylthiouracil against lipid peroxidation caused by potassium bromate in the thyroid gland. J. Cell Biochem..

[34] Karbownik M., Stasiak M., Zygmunt A., Zasada K., Lewinski A. (2006). Protective effects of melatonin and indole-3-propionic acid against lipid peroxidation, caused by potassium bromate in the rat kidney. Cell Biochem. Funct..

[35] Karbownik M., Reiter R.J., Garcia J.J., Cabrera J., Burkhardt S., Osuna C., Lewinski A. (2001). Indole-3-propionic acid, a melatonin-related molecule, protects hepatic microsomal membranes from iron-induced oxidative damage: Relevance to cancer reduction. J. Cell Biochem..

[36] Karbownik M., Garcia J.J., Lewinski A., Reiter R.J. (2001). Carcinogen-induced, free radical-mediated reduction in microsomal membrane fluidity: Reversal by indole-3-propionic acid. J. Bioenerg. Biomembr..

[37] Bendheim P.E., Poeggeler B., Neria E., Ziv V., Pappolla M.A., Chain D.G. (2002). Development of indole-3-propionic acid (OXIGON) for Alzheimer’s disease. J. Mol. Neurosci..

[38] Karbownik M., Lewinski A. (2003). Melatonin reduces Fenton reaction-induced lipid peroxidation in porcine thyroid tissue. J. Cell Biochem..

[39] Karbownik M., Lewinski A. (2003). The role of oxidative stress in physiological and pathological processes in the thyroid gland; possible involvement in pineal-thyroid interactions. Neuro. Endocrinol. Lett..

[40] Lewinski A., Karbownik M. (2002). REVIEW. Melatonin and the thyroid gland. Neuro. Endocrinol. Lett..

[41] Bradford M.M. (1976). A rapid and sensitive method for the quantitation of microgram quantities of protein utilizing the principle of protein-dye binding. Anal. Biochem..

[42] FDA https://www.accessdata.fda.gov/scripts/cdrh/cfdocs/cfcfr/CFRSearch.cfm?fr=184.1635.

[43] Bürgi H., Schaffner T.H., Seiler J.P. (2001). The toxicology of iodate: A review of the literature. Thyroid.

[44] IARC https://monographs.iarc.fr/wp-content/uploads/2018/06/mono73-22.pdf.

[45] Toloza F.J.K., Motahari H., Maraka S. (2020). Consequences of Severe Iodine Deficiency in Pregnancy: Evidence in Humans. Front. Endocrinol..

[46] Zimmermann M.B., Jooste P.L., Pandav C.S. (2008). Iodine-deficiency disorders. Lancet.

[47] Karbownik M., Reiter R.J., Garcia J.J., Tan D.X. (2000). Melatonin reduces phenylhydrazine-induced oxidative damage to cellular membranes: Evidence for the involvement of iron. Int. J. Biochem. Cell Biol..

[48] Karbownik M., Reiter R.J., Garcia J.J., Tan D.X., Qi W., Manchester L.C. (2000). Melatonin reduces rat hepatic macromolecular damage due to oxidative stress caused by delta-aminolevulinic acid. Biochim. Biophys. Acta.

[49] Chyan Y.J., Poeggeler B., Omar R.A., Chain D.G., Frangione B., Ghiso J., Pappolla M.A. (1999). Potent neuroprotective properties against the Alzheimer β-Amyloid by an endogenous melatonin-related indole structure, indole-3-propionic acid. J. Biol. Chem..

[50] Candeias L.P., Folkes L.K., Porssa M., Parrick J., Wardman P. (1995). Enhancement of Lipid Peroxidation by Indole-3-Acetic Acid and Derivatives: Substituent Effects. Free Radic Res..

[51] Zetner D., Andersen L.P.K., Alder R., Jessen M.L., Tolstrup A., Rosenberg J. (2021). Pharmacokinetics and Safety of Intravenous, Intravesical, Rectal, Transdermal, and Vaginal Melatonin in Healthy Female Volunteers: A Cross-Over Study. Pharmacology.

[52] Andersen L.P.H., Werner M.U., Rosenkilde M.M., Harpsøe N.G., Fuglsang H., Rosenberg J., Gögenur I. (2016). Pharmacokinetics of oral and intravenous melatonin in healthy volunteers. BMC Pharmacol. Toxicol..

[53] Baltatu O.C., Senar S., Campos L.A., Cipolla-Neto J. (2019). Cardioprotective Melatonin: Translating from Proof-of-Concept Studies to Therapeutic Use. Int. J. Mol. Sci..

[54] Lin L., Huang Q.X., Yang S.S., Chu J., Wang J.Z., Tian Q. (2013). Melatonin in Alzheimer’s disease. Int. J. Mol. Sci..

[55] Romero A., Ramos E., López-Muñoz F., Gil-Martín E., Escames G., Reiter R.J. (2020). Coronavirus Disease 2019 (COVID-19) and Its Neuroinvasive Capacity: Is It Time for Melatonin?. Cell Mol. Neurobiol..

[56] Acuña-Castroviejo D., Escames G., Figueira J.C., de la Oliva P., Borobia A.M., Acuña-Fernández C. (2020). Clinical trial to test the efficacy of melatonin in COVID-19. J. Pineal Res..

[57] Reiter R.J., Abreu-Gonzalez P., Marik P.E., Dominguez-Rodriguez A. (2020). Therapeutic Algorithm for Use of Melatonin in Patients With COVID-19. Front. Med..

[58] Zhang R., Wang X., Ni L., Di X., Ma B., Niu S., Liu C., Reiter R.J. (2020). COVID-19: Melatonin as a potential adjuvant treatment. Life Sci..

[59] Li X., Cao X., Li J., Xu J., Ma W., Wang H., Wang J., Zhang Y. (2020). Effects of high potassium iodate intake on iodine metabolism and antioxidant capacity in rats. J. Trace Elem. Med. Biol..

